# Wearable Multifunctional Sensors for Human Activity Recognition

**DOI:** 10.3390/s26113420

**Published:** 2026-05-28

**Authors:** Lu Zhang, Yi Du, Haolong Li, Shiquan Yan, Quanxing Yao, Chunyu Liu, Yuejun Zhang, Xiaojian Zhu

**Affiliations:** 1 School of Materials Science and Chemical Engineering, Ningbo University, Ningbo 315211, China; zhanglu23@nimte.ac.cn; 2 Ningbo Institute of Materials Technology & Engineering, Chinese Academy of Sciences, Ningbo 315201, China; duyi@nimte.ac.cn (Y.D.); hl78@mail.ustc.edu.cn (H.L.); yanshiquan@nimte.ac.cn (S.Y.); yaoquanxing@nimte.ac.cn (Q.Y.); liuchunyu2024@nimte.ac.cn (C.L.); 3 Zhejiang Key Laboratory of Magnetic Materials and Applications, Ningbo 315201, China; 4 College of Materials Science and Engineering, Zhejiang University of Technology, Hangzhou 310014, China

**Keywords:** wearable sensors, multifunctional fusion, human activity recognition, flexible electronics, signal decoupling

## Abstract

Driven by the profound convergence of the Internet of Things (IoT) and ubiquitous computing, wearable multifunctional sensors have emerged as a key technology for high-precision human activity recognition (HAR). Advancements in novel materials and flexible electronics have propelled the evolution of these sensors, enabling advances in decoupling heterogeneous signals, enhancing system robustness, and expanding environmental perception. This review systematically examines the frontier research on wearable multifunctional sensors for HAR. We provide an in-depth analysis of three core architectural design paradigms: architecture-level integration, which relies on physical spatial isolation for hardware-level signal decoupling; monolithic integration, which strives for extreme spatial compactness and spatiotemporal signal consistency; and the emerging intrinsically multifunctional design, which leverages novel stimuli-responsive materials for the intrinsic orthogonal discrimination of multidimensional signals. Furthermore, we delineate the diverse application scenarios of these highly integrated sensing platforms across medical rehabilitation, sports science, human–computer interaction (HCI), and daily behavior perception. Finally, this article discusses the critical challenges currently confronting this technology and outlines its future development prospects.

## 1. Introduction

The convergence of the Internet of Things (IoT) and ubiquitous computing has established human activity recognition (HAR) as a key technology for digitizing the physical world and facilitating the construction of digital twins, driving innovation in smart healthcare, personalized sports science, and intuitive human–computer interaction (HCI) [1,2]. However, as application scenarios shift from controlled laboratory environments to unconstrained and dynamic real-world settings, traditional single-modal sensing systems struggle to meet the demands of complex human activities [3,4].

Traditional wearable sensing paradigms predominantly rely on discrete single-transduction sensors—exemplified by inertial measurement units (IMUs) and photoplethysmography (PPG)—and are thus inherently limited to capturing only one dimension of human activity [2]. Human activity, by contrast, is an inherently multidimensional process arising from the complex interplay of neural intentions, muscle dynamics, skeletal kinematics, and environmental interactions [5]. This fundamental mismatch means that the fragmented, partial information yielded by single-modal sensors cannot support either nuanced analysis of activity intentions or comprehensive assessment of holistic health status. When deployed in unconstrained open environments, these limitations are further compounded by inherent perceptual shortcomings: visual occlusion [6], inertial drift [7], and a lack of environmental context, all of which have become critical bottlenecks to reliable performance. While non-contact sensing modalities such as camera-based and radio-frequency (e.g., WiFi)-based systems [8,9] address these issues by enabling ambient intelligence without user compliance, they suffer from fundamental flaws in occluded, out-of-sight, or multi-person scenarios. Most importantly, they cannot directly measure the intimate physiological signals tied to human bodily function, including bioelectric signals and sweat biochemistry. Therefore, non-contact technologies cannot replace on-skin multifunctional sensors. These sensors are essential for collecting high-fidelity, user-specific, and context-rich data, especially in dynamic real-world environments.

**Figure 1:**
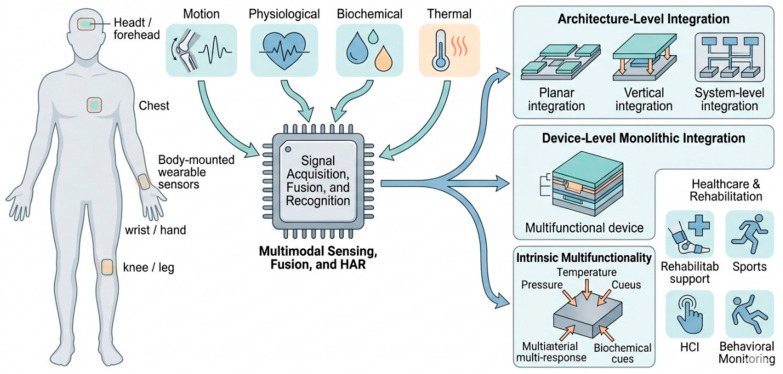
Schematic overview of wearable multifunctional sensors for health monitoring and HAR.

To address these issues, as illustrated in Figure 1, multifunctional fusion—the synergistic integration of heterogeneous data spanning mechanical, kinematic, physiological, and environmental domains—has emerged as an important approach for developing next-generation, robust, and context-aware HAR systems. Its value extends beyond mere signal superposition; rather, it facilitates cross-modal validation and complementary information orchestration to resolve fundamental ambiguities. In recent years, various fusion techniques have demonstrated their potential to tackle specific perceptual challenges. For instance, in medical rehabilitation, fusing electromyography (EMG) with kinematic data enables the precise differentiation between pathological tremors and voluntary movements [10]. In sports science, continuous longitudinal monitoring of physiological load relative to exercise intensity can provide early warnings regarding overtraining [11]. In HCI, integrating strain sensing with inertial data elucidates kinematic synergy patterns, facilitating the reconstruction of virtual grasping intents even under visual occlusion [12]. Furthermore, in ubiquitous computing, fusing environmental context with behavioral data reduces semantic ambiguity, significantly enhancing the robustness of recognizing complex activities of daily living (ADL) [13].

In light of the above, this review proposes a three-tier classification framework for multifunctional integration strategies, systematically distinguishing architecture-level integration, device-level monolithic integration, and material-level intrinsically multifunctional design, and elucidating their respective signal decoupling mechanisms and performance trade-offs. Unlike existing reviews that focus separately on sensor materials or recognition algorithms, this work constructs a full-chain analytical framework spanning hardware integration, multimodal data processing, and scenario-specific human activity recognition (HAR). We further delineate the tailored performance requirements of these sensing platforms for medical rehabilitation, sports science, human–computer interaction (HCI), and daily behavior perception.

## 2. Strategies for Multifunctional Integration

The architectural evolution of wearable multifunctional sensors is driven by the synergistic demands for device miniaturization, mechanical conformability, and spatiotemporal signal consistency [14]. Unlike traditional unimodal sensing designs, multifunctional architectures must fundamentally resolve the inherent trade-off between high integration density and inter-signal cross-sensitivity [15]. To systematically clarify the technical characteristics and performance boundaries of different integration paradigms, we classify current mainstream sensing architectures into three categories: architecture-level integration, device-level monolithic integration, and material-level intrinsically multifunctional design. The core differences among these three strategies are comprehensively compared in Table 1, covering their design philosophies, technical implementations, and key performance trade-offs. In the subsequent subsections, we will elaborate on each integration strategy in detail combined with representative research cases.

### 2.1. Architecture-Level Integration

In the advancement of wearable multifunctional flexible electronics, architecture-level integration has provided a reference for high-fidelity, large-scale array sensing by achieving robust signal decoupling and reducing cross-sensitivity through physical spatial isolation [16]. Its core philosophy is “Physical Isolation” wherein units sensitive to distinct physical stimuli (e.g., pressure, temperature, and bioelectricity) are structurally separated via spatial distribution [17,18], vertical stacking [19,20], or system-level packaging [21]. This approach reduces the propagation paths of crosstalk at the source. It endows the system with the capability to detect multiple stimuli independently and permits the independent optimization of sensitive materials and microstructures for specific physical quantities, thereby achieving precise sensing characterized by high linearity with reduced reliance on complex algorithmic decoupling [22,23].

#### 2.1.1. Lateral Integration

**Figure 2:**
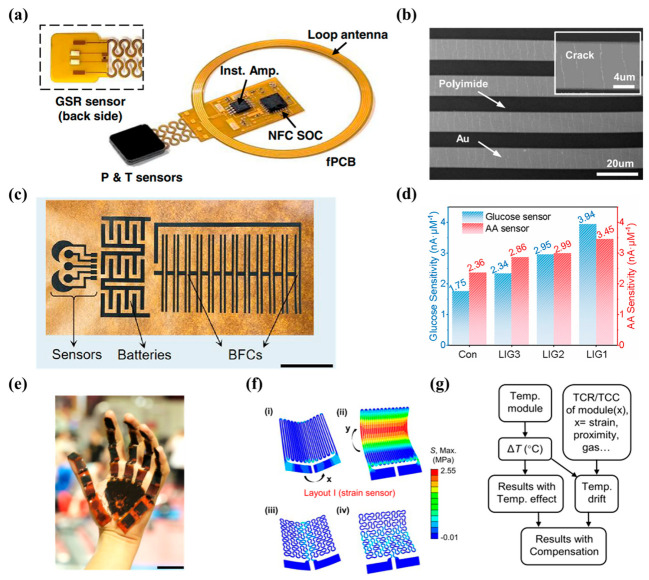
Lateral Integration. (**a**) Double-sided battery-free flexible circuit board; (**b**) Nanoscale cracks on metal film (Reprinted with permission from [16] © Springer Nature, 2023); (**c**) Images of the LIG system electrode; (**d**) Sensitivity contrast of glucose sensors and AA sensors using different electrode materials (Reprinted with permission from [24] © American Chemical Society, 2026); (**e**) Double-sided human-ambience interface; (**f**) Simulation of strain sensitivity control; (**g**) Flowchart of temperature drift compensation mechanism (Reprinted with permission from [25] © American Chemical Society, 2022).

Lateral integration represents the most intuitive and fundamental design paradigm within the architecture-level integration architecture of flexible multifunctional sensors. Its core concept involves the coplanar arrangement or spatial distribution of multiple sensing units, each sensitive to distinct physical or chemical stimuli, onto a single ultrathin flexible substrate. Adhering to the core philosophy of “physical isolation”, this architecture effectively severs the signal crosstalk pathways between different modalities directly at the hardware source, utilizing either spatial distribution within a two-dimensional plane or innovative double-sided layouts. Consequently, this approach enables the independent perception and in situ extraction of multiple parameters without relying on complex backend algorithmic decoupling. Cho et al. [16] proposed a battery-free, multifunctional flexible sensing system employing serpentine interconnects (Figure 2a). To overcome the intrinsically low sensitivity of conventional metal film pressure sensors, the researchers fabricated nanoscale cracks on a Cr/Au metal film via a pre-stretching process and innovatively deposited a nanoscale Parylene-C encapsulation layer on top (Figure 2b). This design not only substantially enhanced the sensor’s sensitivity within the sitting-related pressure range (<100 kPa) but also significantly minimized signal hysteresis, ensuring high linearity and long-term durability. At the hardware level, this multifunctional sensor achieved high conformal integration on an ultrathin flexible printed circuit board (FPCB): a crack-activated pressure sensor and an NTC thermistor for temperature measurement were integrated on the front side, while metal pads serving as a galvanic skin response (GSR) sensor were integrated on the back to evaluate skin hydration levels via sweat. Liang et al. [24] developed a self-powered multimodal wearable sensing system based on an all-in-one integration strategy. Regarding the integration architecture, the system employs laser-induced graphene (LIG) technology to realize the planar, coplanar integration of three functional modules—multimodal sensing, energy storage, and energy harvesting—onto a single polyimide flexible substrate, thereby circumventing the bulkiness and signal interference issues associated with external wiring interconnections in conventional discrete-module assemblies (Figure 2c). This highly integrated planar architecture delivers multiple performance improvements. First, different electrode regions operate cooperatively on the same substrate, achieving excellent intermodule compatibility. Second, the synergistic design of working and counter/reference electrodes enables high sensitivities (3.94 nA μM^−1^ for glucose and 3.45 nA μM^−1^ for ascorbic acid) and favorable selectivity (Figure 2d). Third, the system records ECG and EMG signals with quality comparable to commercial Ag/AgCl gel electrodes. Finally, it retains stable electrochemical sensing and power supply performance after repeated bending and twisting cycles. These results confirm that the integrated architecture reduces system complexity, enhances operational stability, and improves signal acquisition reliability. Wang et al. [25] proposed a double-sided wearable multifunctional sensing system designed for a “human-ambience” interface. Structurally, the system innovatively adopted a double-sided layout, which not only maximized functional density within a limited 2D lateral space but also physically isolated physiological monitoring (facing the human skin) from environmental perception (facing outward) (Figure 2e). To further decouple different physical parameters, the researchers precisely customized the geometric patterns and micro-morphology of the sensing electrodes and modified the active materials, successfully controlling the strain sensitivity of each system module (Figure 2f). Moreover, the implementation of a temperature drift compensation mechanism and the optimization of sensing mechanisms ensured the thermal stability and anti-interference capability of the entire flexible system in complex environments. This anti-interference design, based on customized electrode patterns and double-sided spatial isolation, establishes an engineering approach for constructing highly integrated flexible multifunctional sensors with low crosstalk (Figure 2g).

#### 2.1.2. Vertical Integration

**Figure 3:**
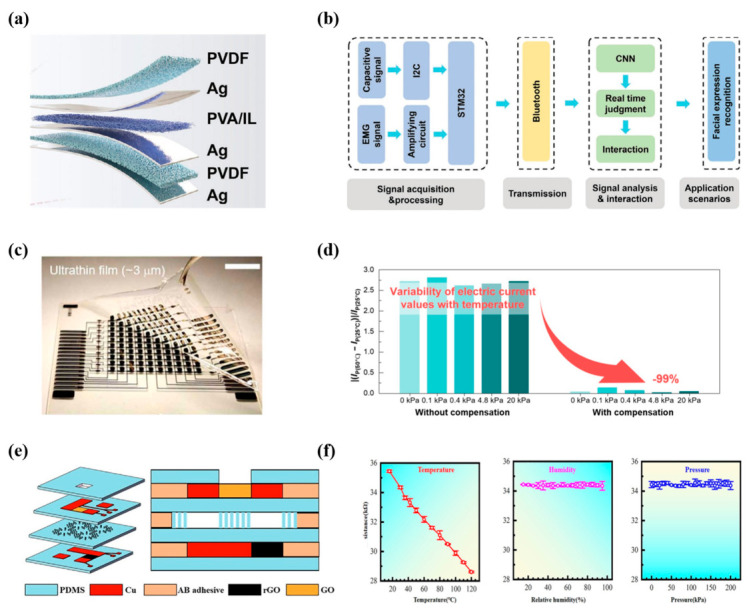
Vertical Integration. (**a**) 3D-stacked capacitive-electromyographic bimodal sensor; (**b**) Facial expression recognition processing pipeline (Reprinted with permission from [26] © John Wiley & Sons, Inc., 2024); (**c**) 3D active-matrix pressure-temperature dual-mode sensor array; (**d**) Effect of thermal distortion compensation mechanism (Reprinted with permission from [27] © American Association for the Advancement of Science, 2025); (**e**) Crosstalk-free hybrid integrated sensors; (**f**) Temperature, humidity, and pressure response curves (Reprinted under a Creative Commons Attribution 4.0 International License from [23] © Elsevier, 2024).

Vertical stacking is a three-dimensional design paradigm for architecture-level integration of flexible multifunctional sensors. It aims to overcome the spatial limitations of 2D planar designs and achieve higher integration densities. At its core, this approach leverages micro/nanofabrication or layer-by-layer assembly techniques to vertically stack functional sensing layers corresponding to distinct physical quantities. To mitigate signal crosstalk, insulating layers or physical isolation barriers are typically interleaved between these heterogeneous strata. This architectural design not only substantially minimizes the physical footprint of the device but, more crucially, the co-location of sensing elements within the 3D space helps to maintain the spatiotemporal consistency of the acquired multidimensional signals, thereby enabling improved in situ synchronous detection [17]. Gao et al. [26] proposed a biocompatible capacitive-electromyographic dual-mode sensor (CEDS) based on a 3D stacking fashion. The upper layer of the device utilizes a spindle-knotted electrospun membrane doped with ionic liquids and a porous electrode to construct a highly sensitive capacitive pressure sensing unit based on the electrical double-layer (EDL) effect. The bottom electrode, directly plated with silver on the skin-attached side, serves as an irritation-free and dehydration-resistant dry electrode for stable surface electromyography (SEMG) monitoring (Figure 3a). By synchronizing hardware-level signal acquisition with a 1D convolutional neural network (1D-CNN) for multifunctional data fusion, this system effectively overcomes the limitations of single-sensor feature extraction and environmental interference (Figure 3b). However, in the arrayed monitoring of pure physical signals (e.g., pressure and temperature), environmental thermal cross-talk poses another severe challenge. To address this, Jo et al. [27] developed a 3D active-matrix multifunctional sensor array based on a vertical integration architecture. To resolve the severe cross-talk between pressure and temperature signals caused by the thermal dependence of charge transport in traditional thin-film transistors (TFTs), the researchers innovatively stacked a TFT pressure sensing unit integrated with an interlocked piezoresistive sheet vertically on top of a TFT temperature sensing unit, sharing a common gate electrode between the two layers (Figure 3c). This design not only achieves highly sensitive and independent perception within a pressure range of 0–20 kPa and a temperature range of 25 °C to 50 °C but also significantly simplifies the wiring and interconnection complexity via the in situ vertical structure. For system-level signal decoupling, a calibrated compensation mechanism was introduced: linear temperature data extracted in situ from the bottom sensor is used to correct the thermal distortion in the output current of the top pressure sensor in real time (Figure 3d). To further resolve the issues of complex integration processes and severe signal cross-talk in multi-parameter epidermal monitoring, Mu et al. [23] proposed a cross-talk-free hybrid integrated multifunctional flexible sensor system. Utilizing solely graphene derivatives (rGO and GO) as sensitive materials, the device was constructed through simple vertical lamination and spraying processes, incorporating a PDMS dielectric layer with circular radiate-shape microstructures (Figure 3e). This specific design not only enhances the pressure sensor’s sensitivity by nearly eight times but also releases residual stress near the electrodes to prevent inter-array cross-talk. It helps each sensor respond primarily to its specific stimulus, achieving effective multifunctional signal decoupling with less dependence on complex algorithms (Figure 3f).

#### 2.1.3. System-Level Integration

**Figure 4:**
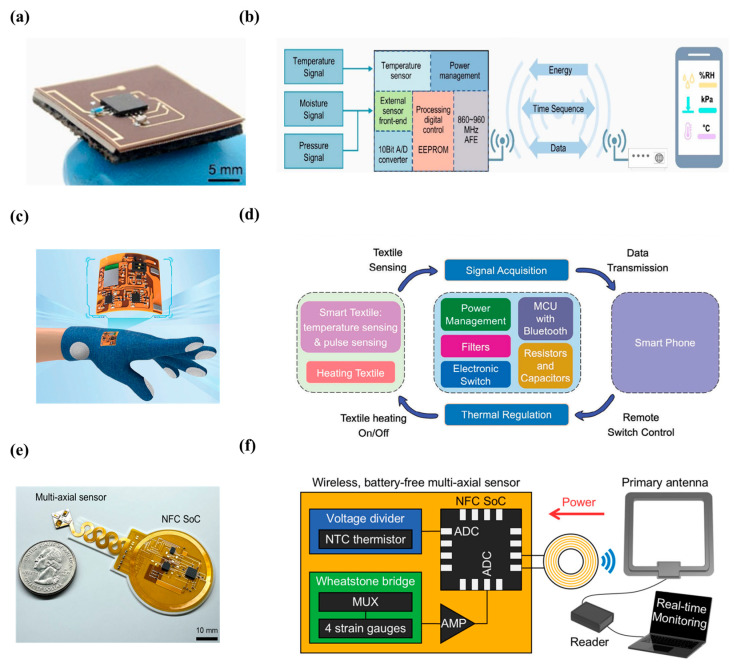
System-Level Integration. (**a**,**b**) Fully integrated multiparameter passive wireless sensor (Reprinted with permission from [29] © American Chemical Society, 2024); (**c**,**d**) Wireless closed-loop system for health monitoring and personalized thermoregulation (Reprinted with permission from [30] © John Wiley & Sons, Inc., 2024); (**e**,**f**) Battery-free multi-axial force sensor with NFC communication (Reprinted under a Creative Commons Attribution 4.0 International License from [31] © Springer Nature, 2025).

Transcending the boundaries of individual sensor devices, system-level integration represents an advanced design paradigm that synergistically combines power sources (e.g., batteries), signal processing circuits, wireless communication modules, and multifunctional sensors into a complete, standalone functional system. By integrating energy harvesting, edge artificial intelligence (AI), and closed-loop control mechanisms onto a single flexible substrate, this architecture transforms traditional passive sensing elements into more autonomous IoT nodes. Ultimately, system-level integration demonstrates considerable potential in enhancing the engineering practicality, environmental robustness, and interactive dimensions of flexible wearable devices for real-world unconstrained applications [28]. Zhou et al. [29] reported a fully integrated multiparameter passive wireless sensor (MWS) for long-term healthcare monitoring (Figure 4a). By innovatively constructing a mechanical–electrical dual-gradient porous structure through a gradient reduction process on a PDMS sponge coated with reduced graphene oxide, they established two independent sensing paths for interlayer resistance and capacitance within a single unit, achieving 2.6 times higher pressure sensitivity than single-gradient designs and a 5-tier moisture detection capability. To bridge the gap between high-precision multiparameter sensing and wireless communication, the MWS was integrated with RFID technology. This integration enables battery-free, simultaneous, and continuous monitoring of pressure, moisture, and temperature across multiple body sites. It also provides a novel platform for wireless body area sensor networks and delivers valuable quantitative insights for personal healthcare management (Figure 4b). In a complementary line of research that extends wearable systems toward closed-loop intervention, Zhang et al. [30] developed a smart textile-driven wireless closed-loop system for decoupled multifunctional health monitoring and personalized thermoregulation (Figure 4c). By designing a thermoresistive fiber with a near-zero thermal expansion coefficient, they achieved precise decoupling of temperature and pressure signals. The integration of this sensory fiber with carbon fiber heaters and a miniature flexible control patch enabled a fully autonomous “sense-decide-act” closed-loop system for maintaining human thermal comfort (Figure 4d). Han et al. [31] proposed a wireless, battery-free multi-axial skin sensor integrated with augmented reality (Figure 4e). Utilizing a near-field communication (NFC) system-on-chip (SoC), this research realized the passive wireless transmission of a flexible, miniaturized multi-axial force sensor, while introducing deep neural networks (DNNs) for the precise calibration and decoupling of complex mechanical data (Figure 4f). Coupled with an AR platform, the system intuitively projected real-time, 3D vector visualizations of skin-interface stresses onto the medical staff’s field of view, significantly enhancing the immersive interactive experience in clinical scenarios such as pressure injury prevention. Thus, through the interdisciplinary integration of energy harvesting, material-level signal decoupling, edge artificial intelligence (AI), and AR visualization technologies, these studies illustrate the potential of system-level integration in improving the practicality, robustness, and interactive dimensions of flexible wearable devices.

### 2.2. Device-Level Monolithic Integration

To pursue higher integration density, sensitive units corresponding to different physical quantities (e.g., piezoresistive, thermoelectric, capacitive) are monolithically manufactured on substrates or chip levels via micro-nanofabrication processes [32,33]. Alternatively, intricate mechanical microstructures (e.g., micro-pillars [34], pores [35], cracks [36], wrinkles [37]) are employed to transduce distinct mechanical stimuli (pressure, shear, strain) into unique variation patterns of conductive pathways or dielectric properties [38,39]. This strategy constructs a hardware foundation for synchronous multiplexed perception at the physical root. While this “all-in-one” strategy imposes stringent challenges on design and fabrication, it yields notable advantages in spatial compactness, spatiotemporal signal consistency, and scalable manufacturing [40].

**Figure 5:**
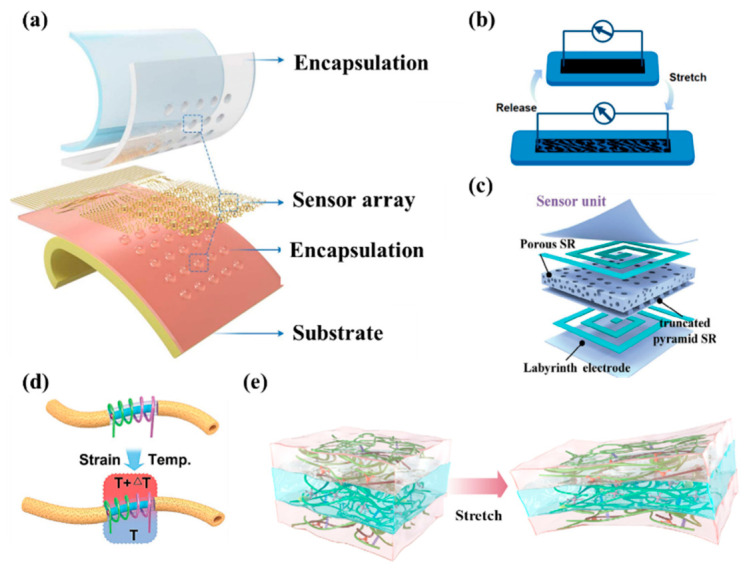
(**a**) 3D biomimetic electronic skin (Reprinted with permission from [21] © American Association for the Advancement of Science, 2024); (**b**) Bionic octopus architecture sensor (Reprinted with permission from [41] © Elsevier, 2024); (**c**) Single-capacitance-mechanism sensor (Reprinted with permission from [38] © John Wiley & Sons, Inc., 2023); (**d**) Heterogeneous material composite dual-mode sensor (Reprinted with permission from [42] © John Wiley & Sons, Inc., 2023); (**e**) Integrated sandwich-structured hydrogel (Reprinted with permission from [43] © John Wiley & Sons, Inc., 2025).

In terms of vector perception, Liu et al. [21] proposed a novel three-dimensionally (3D) architected biomimetic electronic skin. By employing microfabrication techniques and mechanically guided assembly, the researchers densely integrated force sensors (positioned on eight-armed cage mesostructures with a height of ~600 µm, mimicking Merkel cells) and strain sensors (located on arch mesostructures with a height of ~250 µm, mimicking Ruffini endings) into a single flexible device (Figure 5a). This integrated hardware scheme, which combines multiscale 3D microarchitectures with a heterogeneous encapsulation strategy (precisely filling polymers of varying elastic moduli), not only endows the device with multilayered mechanical characteristics similar to human skin but also reduces strain interference at the architectural level, achieving largely decoupled sensing of normal force, shear force, and tensile strain. This approach deeply integrates multiple physical detection mechanisms into a single micro-architected array, and is further combined with system-level deep learning integration. It addresses a key bottleneck of traditional sensors: the inability to simultaneously perform super-resolution tactile localization and quantitative identification of object properties (e.g., elastic modulus and curvature) under flexible deformation. Similarly, Ruan et al. [41] developed a highly adaptable “bionic octopus” architecture. They fabricated the sensor on a fully flexible Ecoflex substrate, integrating two resistive sensing elements in a bionic octopus design. A CNT/Ecoflex conductive elastomer was cast in the central region to form the octopus “body”, which detects normal (vertical) pressure via changes in carbon nanotube density under compression. Eight LIG/Ecoflex strip arrays were radially arranged around the central region to form the octopus “tentacles”, which identify the magnitude and direction of tangential (frictional) force using the microcrack expansion effect under stress (Figure 5b). This monolithic integration design of resistive mechanical sensing units with different dimensions, aided by the joint calibration and mapping of eight-channel electrical signals via a multilayer perceptron (MLP) neural network, effectively mitigates the severe coupling problem of 3D spatial force signals. This highly sensitive, fully resistive integrated scheme was ultimately attached to fingertips as a wearable device, realizing the real-time extraction and 3D reconstruction of meticulous force features during complex handwriting interactions.

Regarding multi-physics perception, Liu et al. [38] proposed a dual-mode flexible sensor based on a single capacitive mechanism. Through pure structural fine-tuning within a single physical architecture, the researchers achieved high-precision decoupling and enhancement of touchless (proximity) and tactile (contact) signals. On the lateral architecture, abandoning traditional parallel plates, they innovatively etched “labyrinth-patterned electrodes”, which significantly enhanced the fringing electric field effect by increasing the physical length of the electrode edges, fundamentally amplifying the easily overlooked weak non-contact signals from the physical level (Figure 5c). On the vertical architecture, a heterogeneous hierarchical dielectric layer of “porous silicone rubber + truncated pyramid” was integrated, utilizing the rapid deformation of the porous structure under low pressure and the continuous response of the pyramid structure under high pressure to achieve highly sensitive pressure sensing over a broad range of 0–200 kPa. This scheme of deeply integrating lateral electrode topological optimization and vertical dielectric layer microstructures within a single flexible capacitor reduces the hardware crosstalk caused by mixing multiple sensing principles. Through backend time-domain signal peak-valley analysis, the system realized switching and real-time monitoring between 3D spatial touchless gesture trajectory recognition and contact pressing actions in one system, effectively enabling the complex interactive control of a robotic arm. Regarding material-level integration, Xiao et al. [42] proposed a sensing architecture with high stimulus discriminability. By integrating sensitive materials or micro-nanostructures with distinct response mechanisms onto a single flexible substrate, the researchers designed the system to treat the easily confounded “strain” and “temperature” sensing mechanisms as “co-framed but independent” in the physical dimension (Figure 5d). This integrated sensing scheme based on heterogeneous material composites reduces crosstalk between physical quantities directly from the hardware capture end, achieving independent, high-precision, and high-resolution separation of mechanical tensile strain and thermal variations with reduced need for extensive backend algorithmic compensation. This design deeply integrates environmental states and human body dynamics into a single micro-device, effectively overcoming the defect of traditional sensors being “difficult to self-discriminate” when facing complex stimuli. Li et al. [43] proposed an integrated sandwich-structured hydrogel architecture. Abandoning the traditional “layer-by-layer stacking + physical adhesion” manufacturing process, the researchers, through a specific crosslinking strategy, in situ constructed connected conductive layers and dielectric/electrolyte layers within a single hydrogel system, forming a microscopic network that is structurally continuous but functionally stratified (Figure 5e). This hardware scheme, which deeply fuses multifunctional sensing units and supercapacitor energy storage units from the fundamental material level, reduces interlayer interface impedance fluctuations and stress concentrations, greatly enhancing the electrical stability of the device during dynamic interactions, and providing an approach to addressing the long-standing system-level challenge of balancing sensing and self-powering in flexible wearable devices.

### 2.3. Material-Level Intrinsically Multifunctionality

This represents an emerging frontier, aiming to explore novel stimuli-responsive active materials (e.g., graphene [44], MXene [45], conductive polymer hydrogels [46], liquid metal composites [47]) that achieve intrinsic multifunctionality at the material level. These materials typically possess tunable electronic band structures or ion-electron coupled conduction characteristics, such that their impedance or potential is simultaneously modulated by strain, temperature, humidity, or biochemical markers (e.g., pH) [48,49]. By elucidating the underlying physicochemical mechanisms (e.g., carrier mobility modulation, ion redistribution, electric double-layer perturbation) and employing advanced signal deconvoluting strategies, it becomes possible to extract multiple target parameters from a single superimposed response signal. This approach represents the evolution of flexible electronics toward greater miniaturization and bio-mimetic perception.

**Figure 6:**
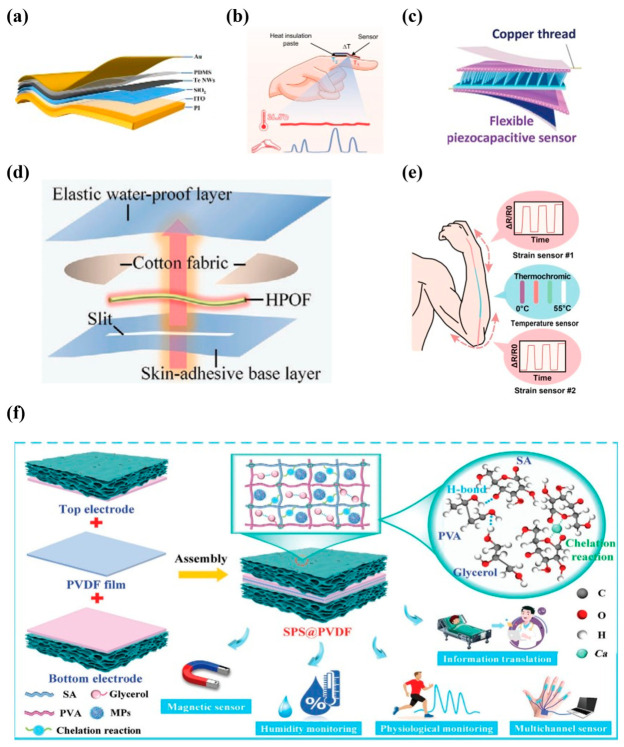
(**a**) Single-layer sensor based on Te nanowires (Reprinted under a Creative Commons Attribution 4.0 International License from [50] © Springer Nature, 2025); (**b**) Self-powered dual-mode sensor (Reprinted under a Creative Commons Attribution 4.0 International License from [51] © Springer Nature, 2025); (**c**) Fabric capacitive sensor (Reprinted with permission from [52] © John Wiley & Sons, Inc., 2025); (**d**) Multi-wavelength orthogonal spectroscopy single optical fiber sensor (Reprinted with permission from [53] © John Wiley & Sons, Inc., 2025); (**e**) Core–shell hybrid fiber sensor (Reprinted with permission from [54] © American Chemical Society, 2020); (**f**) Porous conductive hydrogel sponge (Reprinted with permission from [55] © John Wiley & Sons, Inc., 2024).

Zeng et al. [50] proposed an innovative solution relying on a single active layer of tellurium (Te) nanowires (Figure 6a). By designing a tilt-grown reticulated nanowire structure, they synchronously excited and collected the intrinsic thermoelectric and piezoelectric signals of Te in the out-of-plane direction within a single-channel electrode. This approach leverages the fundamental difference between the direct current (DC) signals generated by the thermoelectric mechanism and the alternating current (AC) pulse signals from the piezoelectric mechanism, enabling the straightforward differentiation of temperature and strain/strain rate signals upon their natural superposition. Similarly, Yang et al. [51] developed a self-powered sensor based on 3D porous laser-induced graphene (LIG) foam nanocomposites incorporated with PEDOT:PSS (Figure 6b). This method overcomes some structural limitations of conventional multi-parameter sensors that rely on complex array stitching. It deeply explores the intrinsic orthogonality of porous graphene’s thermoelectric and piezoresistive effects. Specifically, tensile strain has a negligible impact on thermoelectric voltage, while temperature stimuli barely affect the device’s resistance. By independently measuring these two non-interfering electrical parameters, the single-unit sensor achieved a gauge factor (GF) of 1401.5 and a high temperature resolution of 0.5 °C, significantly reducing device complexity and cost.

Regarding the coupling of microstructures and multi-physical field responses, Song et al. [52] proposed a fabric-based multifunctional flexible capacitive sensor (MFCS) (Figure 6c). Discarding complex multi-layer stacking, they developed a single-sided integrated fabric electrode with a magnetic tilted micropillar (MTM) array. This single architecture deeply couples the material’s magnetic responsiveness with specific microscopic geometries, allowing the device to generate distinctive capacitive responses to pressure (micropillar deformation), spatial proximity (fringing electric field perturbation), and magnetic fields (magnetoelastic bending) under the same capacitive sensing mechanism. This design demonstrated the ability to discriminate multiple physical fields with reduced interference while retaining the flexibility and wearability of the fabric.

In addition to pure electrical decoupling, multi-wavelength and electro-optical orthogonal mechanisms offer novel perspectives for multidimensional signal discrimination. Guo et al. [53] utilized a hydrogel-coated PDMS optical fiber (HPOF) as a single core architecture, intrinsically integrating lanthanide-doped upconversion nanoparticles (Ln-UCNPs) with a pH-sensitive fluorescent dye (Figure 6d). This design enables strain, temperature, and pH sensing mechanisms to operate at distinct independent wavelengths within the same optical fiber. Combined with a ratiometric detection strategy, the system captures spectrally resolved multiband emissions to achieve intrinsic self-calibration and precise simultaneous monitoring with low crosstalk. In the realm of hydrogel-based flexible sensing, Chen et al. [54] developed a multifunctional hybrid fiber with a core–shell segmented structure. By employing an orthogonal electro-optical sensing strategy, the fiber uses resistance changes in the inner hydrogel to monitor joint motion and color changes in the outer thermochromic material to visualize skin temperature (Figure 6e). This approach decouples strain and temperature signals while enabling intuitive, real-time visualization of physiological status for smart clothing applications. Fu et al. [55] developed a highly sensitive porous conductive hydrogel sponge composite, which deeply multiplexes the material’s physical properties (Figure 6f). Through separated capacitive and resistive responses, it intrinsically realizes the independent discrimination of magnetic fields, mechanical forces, and humidity stimuli. Building on this high integration, to tackle complex hand gesture interaction scenarios characterized by high nonlinear coupling, the study further introduced a CNN-GRU deep learning algorithm as a “soft decoupling” engine, achieving semantic recognition of high-degree-of-freedom gesture language with an accuracy of 99.17% on a flexible sponge array.

To quantitatively compare the technical specifications and application capabilities of the representative multifunctional sensors discussed in this chapter, we summarize their core performance parameters, integration strategies, and supported HAR tasks in Table 2. This table systematically collates the detection targets, sensitive materials, sensing mechanisms, and recognition accuracy of typical works across the three integration paradigms, providing a clear reference for evaluating the technical maturity and application potential of different design schemes.

## 3. Human Activity Recognition Pipeline for Multifunctional Wearable Sensors

**Figure 7:**
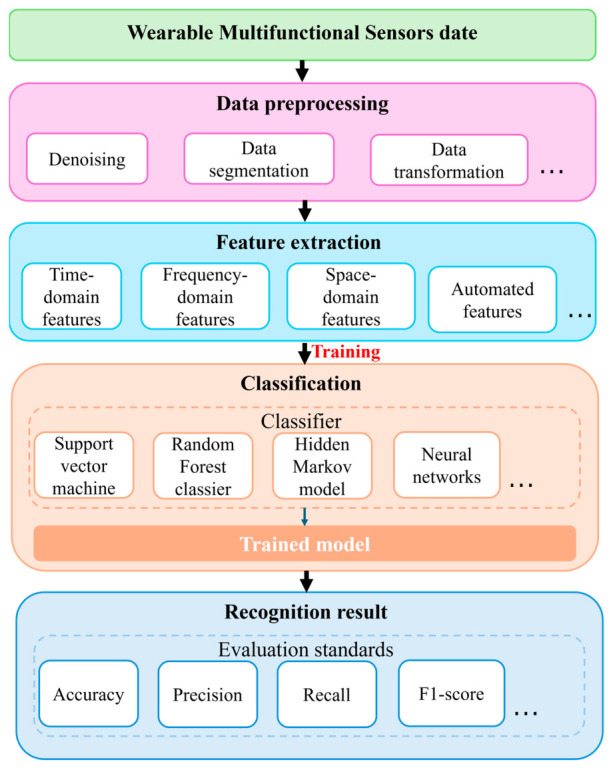
Flowchart of data processing and pattern recognition for wearable multifunctional sensors.

High-performance sensors form the fundamental basis of HAR. Nevertheless, raw sensor data in isolation is insufficient to enable practical ubiquitous deployment and real-world applications. From a computational perspective [56], HAR based on wearable multifunctional sensors is inherently a complex multivariate time-series classification task [57], whose core lies in constructing a standardized data processing pipeline that ultimately outputs definitive activity class labels (Figure 7). Particularly for resource-constrained wearable devices, this pipeline must achieve a delicate balance among recognition accuracy, computational latency, and energy consumption. This chapter systematically elaborates on each core component of this complete processing pipeline.

In terms of data preprocessing, raw sensor data must be preprocessed upon acquisition to provide appropriate input for subsequent activity recognition [58]. Typically, the first step is to remove noises and outliers from the raw sensor streams. To endow continuous and heterogeneous sensor data with explicit behavioral semantics, the pipeline generally relies on the sliding window technique to segment the original time-series signals into fixed-length feature segments. The recognized activities span an extremely wide range of granularities, encompassing simple actions (e.g., walking), complex composite behaviors (e.g., housework), and even extremely brief posture transition phases (e.g., transitioning from walking to standing). Beyond denoising and data segmentation, further preprocessing of sensor data is usually required, such as coordinate system transformation, time-frequency transformation, and color space conversion, to generate suitable representations for activity recognition [59]. Specifically, data transformation for motion sensor data, acoustic signals, and images/videos focuses on coordinate system alignment, frequency-domain analysis, and object extraction, respectively. In terms of computational difficulty, denoising and data segmentation are generally straightforward to implement, whereas complex data transformations are more computationally intensive. Regarding frequency of usage, denoising and data segmentation are adopted in the vast majority of research works, as extracting clean and efficient sensor data corresponding to human activities is an indispensable prerequisite. The adoption of data transformation depends on the requirements of the recognition task and the chosen recognition approach. Typically, knowledge-driven approaches or traditional machine learning-based approaches impose higher requirements on data transformation, while deep learning-based approaches have relatively lower demands.

In terms of recognition approaches, HAR is undergoing a profound paradigm shift from traditional machine learning to deep learning. Commonly used classifiers include Support Vector Machine (SVM) [60], Random Forest (RF) [61], Hidden Markov Model (HMM) [62], and Neural Networks (NN) [63], among others. Early machine learning methods are computationally lightweight but heavily rely on labor-intensive and time-consuming handcrafted feature (time-domain/frequency-domain) extraction based on expert experience. Deep Learning (DL) breaks this bottleneck through automated feature engineering. Among various network architectures, Convolutional Neural Networks (CNN) excel at capturing local spatial dependencies and extracting hierarchical features from multi-sensor data streams; Recurrent Neural Networks (RNN) and their optimized variants (Long Short-Term Memory, LSTM; Gated Recurrent Unit, GRU) are specifically designed for modeling long-range temporal dependencies in time-series signals. Currently, hybrid architectures integrating CNN and LSTM have achieved state-of-the-art performance on mainstream HAR benchmark datasets, as they synergistically combine the strengths of spatial feature extraction and temporal dynamics modeling. To systematically compare the applicability, advantages, and limitations of different classification algorithms for HAR tasks, we summarize their core characteristics and typical recognition accuracy in Table 3.

Nevertheless, the most significant barrier to real-world deployment lies in model generalization capability. Due to the high cost of constructing training datasets with ground-truth labels, model development is highly dependent on high-quality benchmark datasets. Currently, the academic community widely adopts public datasets such as OPPORTUNITY, PAMAP2, WISDM, and UCI-HAR to standardize task definitions and validate algorithm effectiveness [71,72]. However, models that perform excellently on controlled laboratory data often suffer from significant performance degradation when applied to new users in real-world environments. This “domain shift” phenomenon primarily arises from inter-individual physiological differences, sensor placement drift, and irregular behavioral patterns. To improve cross-user generalization and robustness, the academic community has introduced transfer learning mechanisms and data augmentation techniques to expand long-tailed data distributions and reduce reliance on massive labeled data. To accurately evaluate such real-world generalization capability, rigorous experimental protocols such as Leave-One-Subject-Out (LOSO) cross-validation are widely adopted in the field to effectively eliminate subject-specific biases [73]. Meanwhile, in terms of evaluation metrics, given the extreme class imbalance present in real-world HAR scenarios (e.g., fall detection), a single Accuracy metric is highly misleading. Precision, Recall, and the F1-score that harmonizes the two have become indispensable metrics for comprehensively assessing the credibility of model predictions [74].

## 4. Applications of Wearable Multifunctional Sensors for HAR

Compared with wearable unimodal sensors, wearable multifunctional sensors offer distinct advantages for HAR by integrating physiological [4], motion [75], and environmental data [76]. This section overviews the applications of these integrated systems in medical rehabilitation, sports science, HCI, and behavior perception.

### 4.1. Healthcare and Rehabilitation

In medical rehabilitation scenarios, the paramount requirements for wearable multifunctional sensors are quantitative precision, physiological interpretability, and clinical validity. Unlike consumer-grade gadgets, medical-grade wearables must deliver actionable insights that correlate with established clinical standards. For instance, in diabetes management, quantifying multiplexed biochemical biomarkers (e.g., branched-chain amino acids, cortisol, lactate, cytokines) in biofluids provides critical insights into insulin resistance, metabolic stress, and diabetic complications [77].

#### 4.1.1. Chronic Disease Management

**Figure 8:**
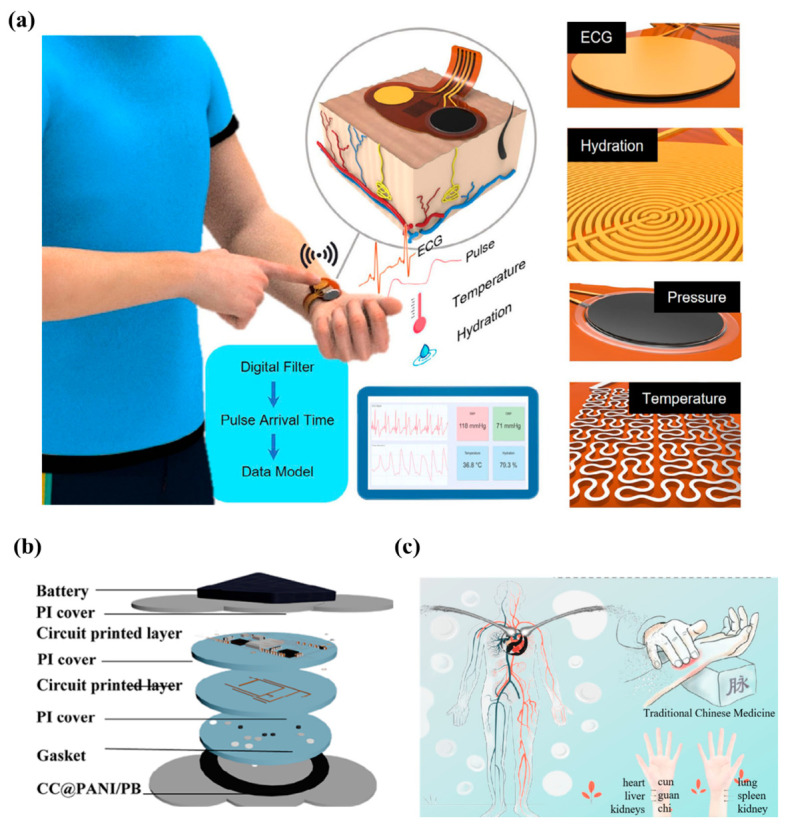
Chronic Disease Management. (**a**) Miniaturized multifunctional epidermal patch (Reprinted with permission from [79] © John Wiley & Sons, Inc., 2025); (**b**) Pomegranate-inspired composite monitoring device; (**c**) Cardiovascular disease prediction via deep learning (Reprinted with permission from [80] © John Wiley & Sons, Inc., 2025).

For patients with chronic conditions (e.g., diabetes, cardiovascular diseases), relying solely on snapshot physiological metrics often lacks contextual relevance, as vital signs are significantly modulated by physical activity. Multifunctional sensors, by integrating activity context with physiological data, enable the precise calibration of pathological assessments [78]. Sharma et al. [79] developed a miniaturized multifunctional epidermal patch that integrates a piezoresistive pulse sensor, a biogel-enhanced ECG sensor, a skin hydration sensor, and a temperature sensor onto a single flexible polyimide substrate for comprehensive cardiovascular health assessment (Figure 8a). By simultaneously acquiring high-quality ECG and radial artery pulse signals from the wrist, the MEP enables cuffless, continuous blood pressure estimation through pulse arrival time extraction combined with a Random Forest Regression model, achieving a bias of 0.59 ± 5.03 mmHg for systolic and 0.68 ± 2.86 mmHg for diastolic blood pressure against a commercial sphygmomanometer. In vivo experiments demonstrated that the MEP not only reliably tracks blood pressure variations during exercise and rest over multiple days, but also captures subtle physiological changes such as vasodilatory and vasoconstrictive temperature responses, post-exercise skin hydration elevation, and heart rate dynamics during physical activity. This highly compact, multifunctional sensing strategy, which synergizes continuous hemodynamic monitoring with skin physiological state tracking, addresses some of the single-parameter limitations of conventional wearable cardiovascular sensors. Consequently, it provides a reliable and unobtrusive hardware platform for early detection of hypertension, exercise-induced cardiovascular assessment, and personalized digital healthcare management. Addressing the bottleneck of long-term energy supply, Zhao et al. [80] developed a flexible, self-powered multifunctional sensing system utilizing a pomegranate-inspired polyaniline-Prussian blue (CC@PANI-PB) composite. This innovative platform merges a photo-assisted lithium-oxygen battery with an ultra-sensitive sensor array capable of simultaneously acquiring mechanical pressure pulse waves and bioelectric electrocardiogram signals (Figure 8b). Exhibiting a high sensitivity of 4.16 × 10^7^ kPa^−1^ and good mechanical durability over 9000 cycles, the tactile sensor precisely captures subtle radial artery pulsations, effectively mirroring Traditional Chinese Medicine (TCM) diagnostic techniques. Crucially, by coupling this dual-mode data acquisition with an integrated deep learning module, the system facilitates real-time pulse wave interpretation and ECG classification, achieving a prediction accuracy of 95.90% for cardiovascular disease. This paradigm of integrating energy autonomy, multifunctional signal acquisition, and artificial intelligence provides a robust hardware foundation for next-generation wearable healthcare monitoring (Figure 8c).

#### 4.1.2. Rehabilitation Assessment

**Figure 9:**
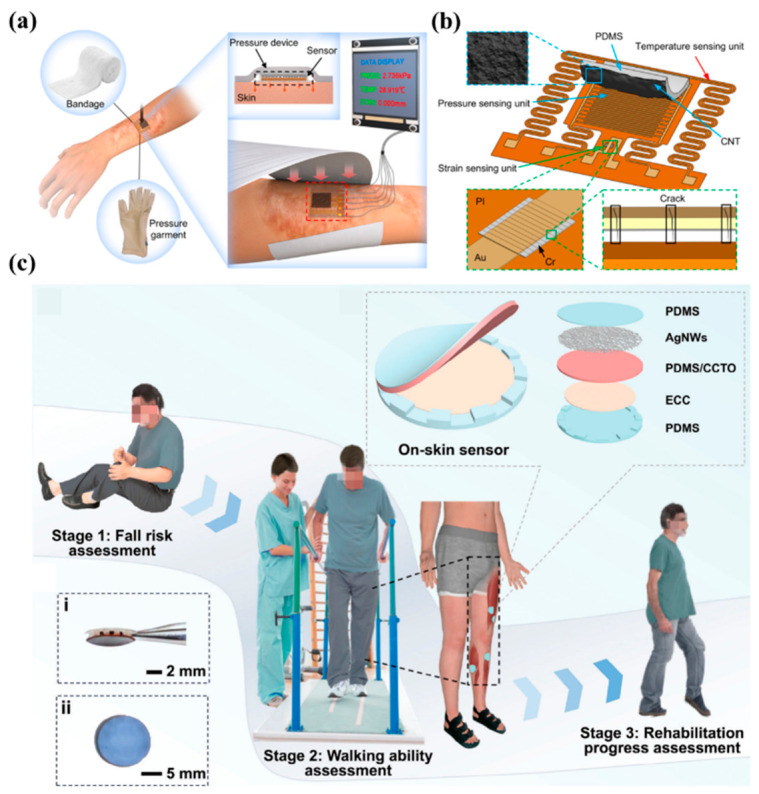
Rehabilitation Assessment. (**a**) Biomimetic spinosum-structured flexible sensor; (**b**) Sensor structure eliminating blind compression interference (Reprinted with permission from [82] © American Chemical Society, 2025); (**c**) Rehabilitation assessment platform combining triboelectric sensors and MG-former (Reprinted with permission from [83] © John Wiley & Sons, Inc., 2024).

In post-operative rehabilitation, objectively quantifying the recovery trajectory of motor functions is critical for tailoring treatment plans. Multifunctional sensors provide granular data on both physiological load and kinematic fidelity [81]. Liu et al. [82] developed a multifunctional, skin-conformal flexible sensor tailored specifically for pressure therapy and motion tracking in burn rehabilitation (Figure 9a). This integrated device uses an epidermis-inspired spinosum microstructure for highly sensitive low-pressure monitoring (35%/kPa within the therapeutic range of 1.3–3.3 kPa). It also incorporates a precisely controlled nanocracking metallic film for strain detection and a serpentine-structured unit for temperature sensing (Figure 9b). Crucially, by demonstrating through simulations and empirical tests that pressure and strain effects are spatially localized while temperature exerts a global influence, the system reduces cross-modal signal interference. In practical HAR applications, this decoupled sensing architecture successfully distinguished therapeutic joint kinematics (e.g., wrist flexion) from adjunctive mechanical actions (e.g., external finger compression), establishing a technical approach for quantitative rehabilitation assessment and complex activity classification in dynamic clinical environments. At the system level, Wang et al. [83] developed a full-process, fine-grained, and quantitative rehabilitation assessment platform (RAP) synergizing flexible on-skin triboelectric sensors with an advanced multi-task gait transformer (MG-former) model. The highly conformable sensor, engineered with a polydimethylsiloxane/CaCu_3_Ti_4_O_12_ (PDMS/CCTO) composite, precisely captures subtle skin deformations induced by lower limb muscle activities during dynamic gait (Figure 9c). Crucially, the integrated MG-former model simultaneously executes binary classification, multiclassification, and regression tasks to sequentially evaluate fall risk, walking ability, and continuous rehabilitation progress. During clinical validation involving post-stroke hemiplegic patients, this platform achieved a high assessment accuracy of 96.18%, demonstrating high consistency with standardized physician evaluations. Notably, the system’s regression-based tracking quantified slight, continuous improvements in patient ambulatory ability that conventional discrete clinical scales could not detect, thereby providing a robust, AI-driven technical foundation for dynamically adjusting personalized rehabilitation strategies.

#### 4.1.3. Elderly Care

**Figure 10:**
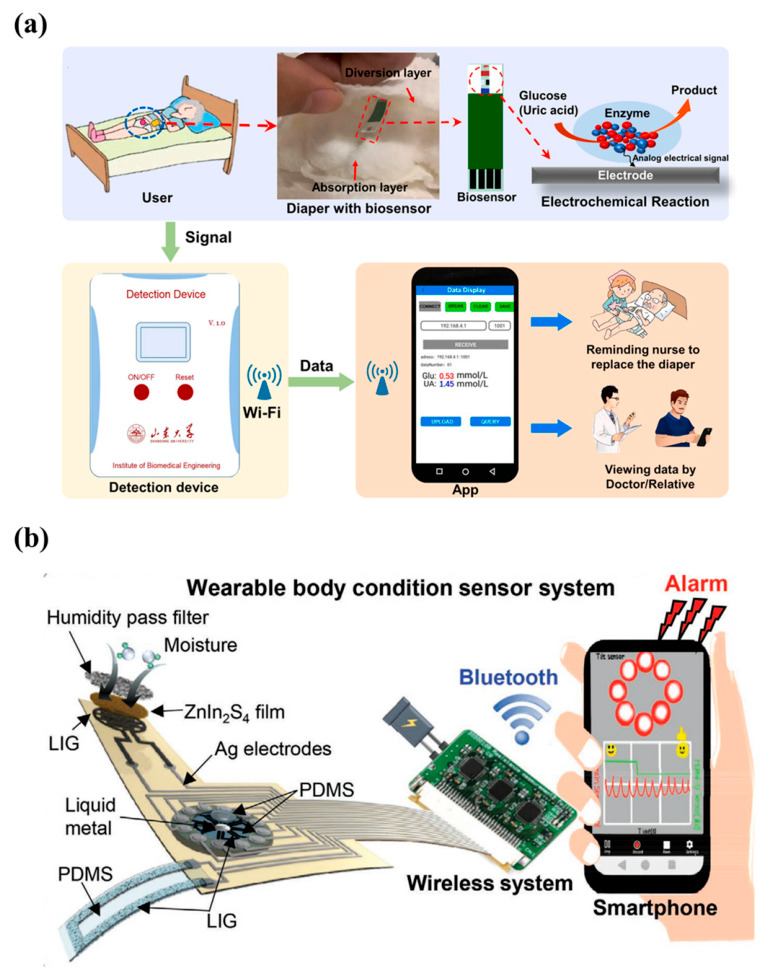
Elderly Care. (**a**) Smartphone-based in situ urine detection system (Reprinted with permission from [85] © Elsevier, 2022); (**b**) Wearable multimodal wireless sensor system for sleep safety monitoring (Reprinted with permission from [86] © John Wiley & Sons, Inc., 2021).

With the intensification of population aging, HAR technologies for the elderly are evolving from simple fall detection to comprehensive physiological surveillance and remote health monitoring [84]. Su et al. [85] engineered a wearable sensing system that integrates a multi-parameter electrochemical biosensor into an ordinary diaper for the real-time detection of urinary glucose and uric acid (Figure 10a). A pivotal innovation of this device is the incorporation of an active self-locking valve utilizing a superabsorbent polymer (SAP). This structural design mitigates the interference of urine volume fluctuations and prevents reverse fluid suction, thereby maintaining a highly stable reaction microenvironment for precise biochemical quantification. Coupled with a portable wireless detection device and a smartphone application, the flexible sensor demonstrated excellent sensitivity and selectivity in dynamic clinical tests. Xu et al. [86] presented a laser-induced graphene (LIG)-based wearable multifunctional wireless sensor system with integrated feedback alarm functions, specifically designed for real-time sleep safety monitoring of the elderly and infants (Figure 10b). By integrating a liquid metal-based tilt sensor capable of distinguishing 18 distinct sleeping postures, a respiration rate sensor, and a diaper moisture sensor onto a single flexible substrate, the system enables comprehensive, non-invasive surveillance of body conditions during sleep. The multi-channel signals are wirelessly transmitted via Bluetooth to a smartphone interface, where pre-programmed alarm thresholds trigger immediate audiovisual alerts upon detecting prone sleeping, respiratory arrest, or excessive diaper wetness. This intelligent closed-loop sensing paradigm, which merges scalable laser-based manufacturing, unobtrusive multifunctional signal acquisition, and timely caregiver notification, helps to bridge the gap between passive monitoring and active emergency intervention. Consequently, it provides a highly reliable, cost-effective, and user-friendly hardware platform. This platform reduces the risk of sudden death syndromes, supports remote geriatric care, and alleviates the persistent burden on caregivers of elderly individuals with limited self-care abilities.

### 4.2. Sports Science

In competitive sports and athletics, traditional motion capture and physiological testing rely heavily on bulky laboratory optical systems and benchtop analyzers, failing to capture in-the-wild performance metrics during actual competitive scenarios [87]. The advent of multifunctional flexible sensors has reduced spatial constraints, enabling the synchronous in-field monitoring of biomechanical kinematics (technical execution) and physiological metabolic load (physical status) [88]. This real-time fusion of multidimensional data is pivotal for scientifically preventing injuries and optimizing competitive strategies.

#### 4.2.1. Motion Analysis

**Figure 11:**
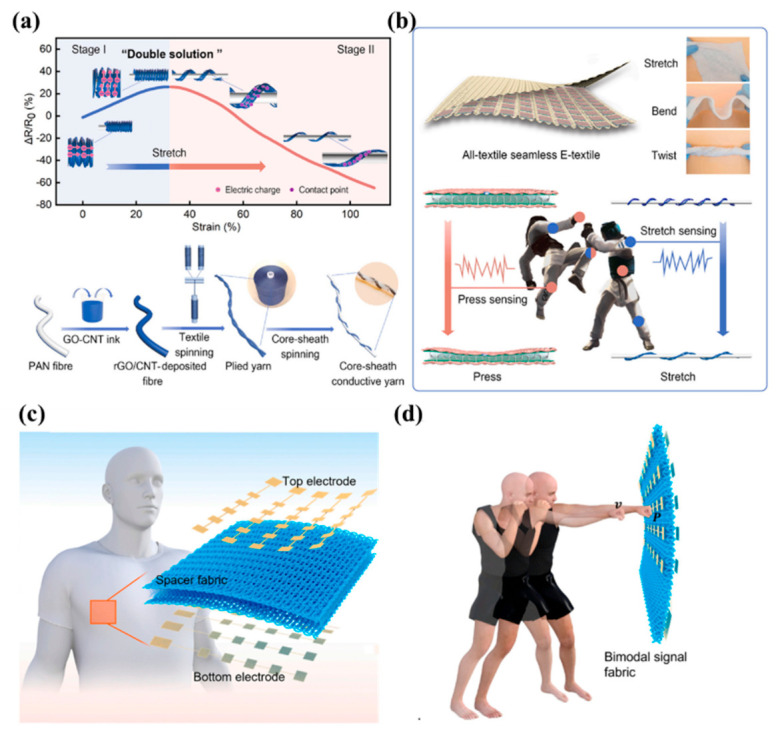
Motion Analysis. (**a**) Schematic of piezoresistive core-sheath yarn and capacitive 3D spacer fabric; (**b**) All-textile bimodal sensor for Taekwondo (Reprinted with permission from [89] © Elsevier, 2021); (**c**) Bimodal all-textile (BAT) capacitive sensor; (**d**) Application of boxing motion monitoring (Reprinted with permission from [90] © Elsevier, 2022).

The design of HAR systems in sports is transitioning from generic activity classification to fine-grained technical analysis specific to sporting disciplines. Addressing the dual challenges of large limb deformation and high-impact forces in Taekwondo, Ma et al. [89] proposed an “all-in-one” seamless electronic textile (E-textile) capable of dual tactile and tension perception. This system intelligently integrates a piezoresistive core-sheath yarn for strain monitoring and a capacitive 3D spacer fabric for pressure sensing. A significant technological bottleneck in traditional helical core-sheath yarns is the nonmonotonic “double solution phenomenon” during large elongation. The researchers reduced this signal ambiguity by applying an insulating polyurethane (PU) coating prior to twisting, thereby achieving a reliable, monotonic strain detection range up to 90%. Exhibiting a robust pressure detection capacity up to 110 kPa and durability across 100,000 cycles, the fully textile-based sensor maintains intrinsic air breathability and washability (Figure 11a). During practical HAR validation, the dual-modal E-textile was embedded into a Taekwondo uniform, successfully decoupling and simultaneously quantifying the dynamic knee flexion (tension) and the localized chest impact (tactile pressure) during simulated combat training. This integration strategy provides a highly durable and comfortable hardware foundation for full-body kinematic analysis and form correction in intense athletic scenarios (Figure 11b). Building on this, to capture dynamic features, Ye et al. [90] developed a bimodal all-textile (BAT) capacitive sensor capable of simultaneous touchless (proximity) and tactile (pressure) detection (Figure 11c). The system ingeniously utilizes a 3D spacer knitted fabric decorated with graphene nanosheets as the dielectric layer, sandwiched between nickel-plated woven fabric electrodes. By exploiting fringe-field interference for touchless induction and parallel-plate distance variation for tactile sensing, the device achieves highly sensitive, synchronized capture of approaching distances (up to 20 cm) and normal pressures. During practical validations in boxing exercises, this bimodal textile sensor was integrated into training garments, decoupling and quantifying the instantaneous punching velocity and impact force in real-time (accurately distinguishing the dynamic characteristics among slow, medium, and quick punches). This “all-textile” design paradigm merging breathability, washability, and touchless/tactile dual-signal acquisition provides a promising hardware solution for multidimensional kinematic parameter decoupling and digitalized training in complex, highly dynamic sports scenarios (Figure 11d).

#### 4.2.2. Injury Prevention

**Figure 12:**
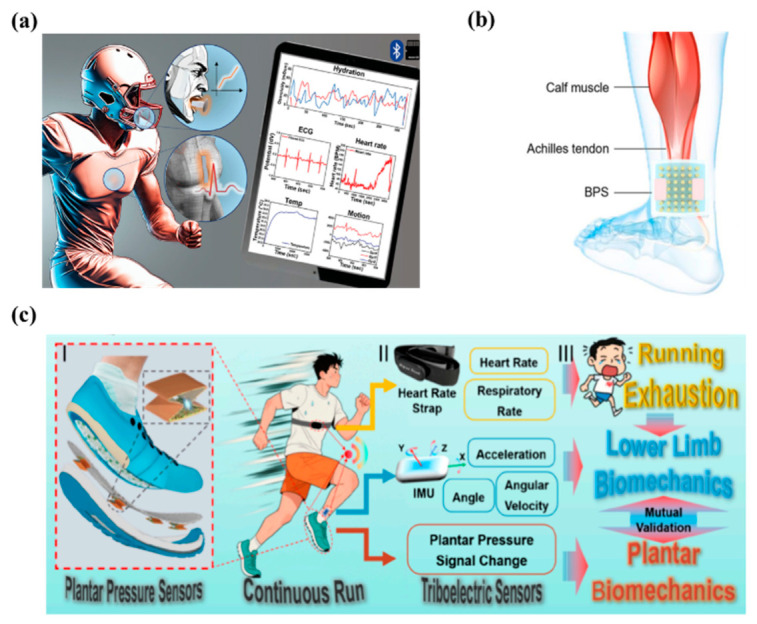
Injury Prevention. (**a**) Wireless monitoring system fusing skin patches and mouthguards (Reprinted under a Creative Commons Attribution 4.0 International License from [11] © John Wiley & Sons, Inc., 2024); (**b**) Bimodal Achilles tendon monitoring piezotronic sensor (Reprinted under a Creative Commons Attribution 4.0 International License from [91] © Springer Nature, 2025); (**c**) Bio-based triboelectric fatigue monitoring system (Reprinted with permission from [92] © American Chemical Society, 2025).

Overtraining and non-functional overreaching often stem from an imbalance between external training load (e.g., distance, pace) and internal physiological load (e.g., heart rate, metabolic stress). Multifunctional sensors can acutely identify fatigue thresholds by synchronizing these data streams. Kim et al. [11] developed an all-in-one, wireless, multi-sensor integrated wearable system specifically engineered for the real-time, continuous detection of dehydration and physiological stress in athletes (Figure 12a). This comprehensive platform synergizes a conformable epidermal soft patch with a uniquely designed sensor-integrated mouthguard to non-invasively and simultaneously monitor multifaceted physiological indicators, including saliva osmolality, skin temperature, and cardiac functions. During dynamic validation, the system captured synchronized acute elevations in dehydration and physiological strain within intensive performance windows. By providing early, quantitative warnings of hidden physical exhaustion and cardiac stress during extreme activities, this multifunctional integrated architecture establishes a technological framework for preventing severe sports-related injuries (e.g., heatstroke and overexertion), thereby extending the utility of HAR systems from mere kinematic tracking to holistic athletic safety management. Focusing on local structural damage prevention, Wang et al. [91] proposed a bimodal piezotronic sensor (BPS) utilizing Y-ion-doped ZnO nanorods, circumventing the structural complexity and signal crosstalk commonly associated with integrating multiple sensing mechanisms (Figure 12b). By leveraging the piezotronic effect to modulate carrier transport via strain-induced piezoelectric potential, this device achieves high responsiveness to both static and dynamic stimuli, with a gauge factor of 23,439 and a stable static force response duration exceeding 600 s. In practical HAR applications, the BPS was deployed to continuously monitor Achilles tendon kinematics under mixed loading conditions. When coupled with a 1D-CNN, the system classified distinct tendon states with 96% accuracy, establishing a highly reliable, threshold-based early warning mechanism to prevent tissue damage during over-strenuous movements. This monolithic bimodal sensing strategy provides a robust technological framework for the real-time evaluation of tissue health and the proactive prevention of localized sports injuries. Liu et al. [92] developed a biobased, self-driven TENG utilizing pulp wool tailored for the multifunctional analysis of exercise fatigue (Figure 12c). By incorporating barium titanate (BaTiO_3_) nanoparticle doping and a unique bidirectional W-shaped encapsulation strategy, the flexible sensor achieved a response time of 8.4 ms and robust mechanical durability over 6200 cycles. Crucially, rather than relying on a single data source, the researchers established a comprehensive multifunctional monitoring system by integrating the self-powered PW-TENG with an inertial measurement unit and a heart rate strap (HRS). During dynamic running experiments across various speeds, this integrated platform simultaneously captured a wide array of kinematic and physiological metrics. By applying Principal Component Analysis (PCA) to the multi-dimensional dataset, the system decoupled and quantified distinct progressive stages of physical fatigue. This innovative paradigm fusing biobased, self-powered triboelectric sensors with multifunctional data analytics provides a sustainable hardware-software framework for daily sports training and dynamic health management.

### 4.3. Human–Computer Interaction

In the realm of HCI, the paradigm is shifting from traditional peripherals (keyboard/mouse) to naturalistic, immersive multifunctional interfaces. Wearable multifunctional sensors, by extending the sensing layer to the human body surface, capture high-fidelity, low-latency intent signals, ultimately forming a closed-loop interaction system [93].

#### 4.3.1. Gesture Recognition

**Figure 13:**
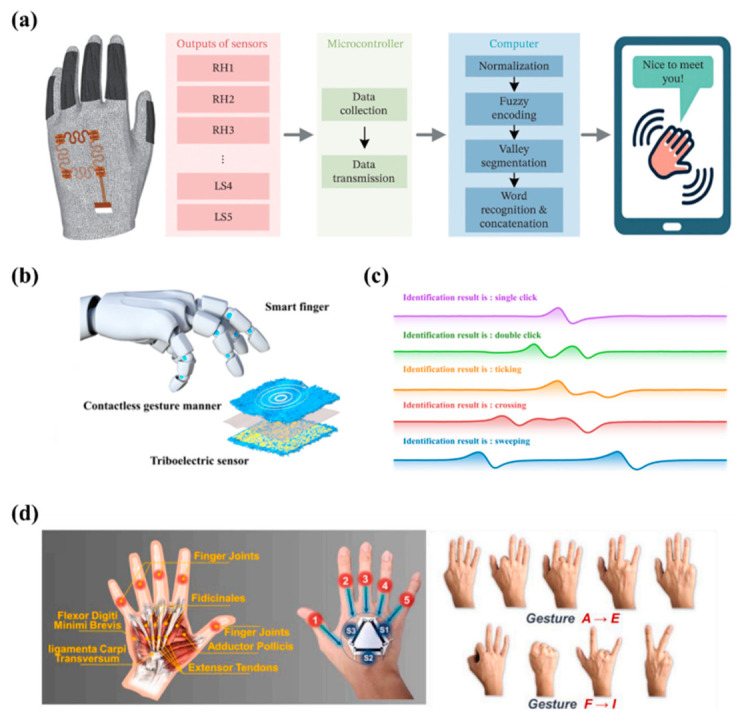
Gesture Recognition. (**a**) Sign language recognition system combining multifunctional sensing and fuzzy encoding (Reprinted under a Creative Commons Attribution 4.0 International License from [94] © John Wiley & Sons, Inc., 2024); (**b**) Bimodal coupled multifunctional haptic perceptron; (**c**) Waveforms of contactless gesture and material identification (Reprinted with permission from [95] © Springer Nature, 2024); (**d**) Paper-based strain sensor array and gesture reconstruction (Reprinted under a Creative Commons Attribution 4.0 International License from [96] © Elsevier, 2024).

The core of constructing wearable systems for complex HCI lies in heterogeneous hardware synergy to overcome the information bottlenecks of single sensing dimensions. Addressing the challenge of reconstructing high-degree-of-freedom (DoF) hand movements, Wei et al. [94] developed a continuous SLR system that synergizes multifunctional hand/finger movement sensing with a fuzzy encoding strategy (Figure 13a). This platform employs stretchable fabric strain sensors to precisely capture discrete finger joint flexions, while integrating IMUs to monitor the global orientation and spatial trajectories of the hand. A key feature of this work is the implementation of a fuzzy encoding module that maps raw sensor data into robust semantic representations, allowing for “data-efficient universal recognition”. Specifically, the model trained on limited word-level samples from a single individual can be successfully generalized to recognize complex sentences performed by new, untrained users. This framework achieves excellent performance, with word-level and sentence-level recognition accuracies of 98.3% and 92.5%, respectively. It represents a significant advance toward low-resource, cross-user gesture recognition, and provides a scalable hardware-software solution for advanced HCI and assistive technologies for the hearing and speech impaired. Ye et al. [95] engineered a bimodal coupled multifunctional haptic perceptron that intelligently integrates capacitive and triboelectric sensing modalities (Figure 13b). A key structural innovation of this device is its symmetrical physical isolation design, which prevents signal crosstalk between the two sensors while utilizing an energy complementarity strategy to reduce overall power consumption. Functionally, the capacitive unit exhibits a broad linear response range (0–745.3 kPa) to precisely quantify contact mechanics, such as material hardness and deformation, whereas the triboelectric unit is highly sensitive to the electron affinity of approaching objects, enabling proximity sensing. Empowered by integrated machine learning algorithms, this bimodal perceptron achieved highly accurate contactless gesture recognition alongside dynamic material identification (Figure 13c). This symmetrical coupling paradigm offers a highly efficient, low-interference hardware solution for next-generation HAR systems, particularly in complex scenarios requiring hybrid (contact and contactless) multidimensional interactions. Adopting a “Less is More” strategy, Lin et al. [96] developed a low-pixel, paper-based strain sensor array capable of multidimensional strain decoupling. Fabricated via a cost-effective inkjet printing technique, the sensitive layer utilizes mechanically induced microcrack structures to precisely distinguish between inward and outward bending strains. More importantly, by arranging only three sensing units in a predefined triangular configuration, the array extracts the horizontal angle of the strain axis. This parameter is critical for decoupling multidimensional surface deformations. Rather than monitoring individual joints, this low-pixel array was deployed on pivotal intersections of human skin (e.g., the dorsum of the hand and the upper back) to capture the synergistic kinematic patterns of underlying muscle-tendon complexes (Figure 13d). Empowered by neural network algorithms, this streamlined architecture achieved 100% accuracy in recognizing both complex hand gestures and diverse upper limb activities in the tested scenarios. This strategy reduces device complexity and hardware redundancy, providing a highly scalable and computationally efficient approach for next-generation wearable motion capture systems.

#### 4.3.2. Virtual Reality

**Figure 14:**
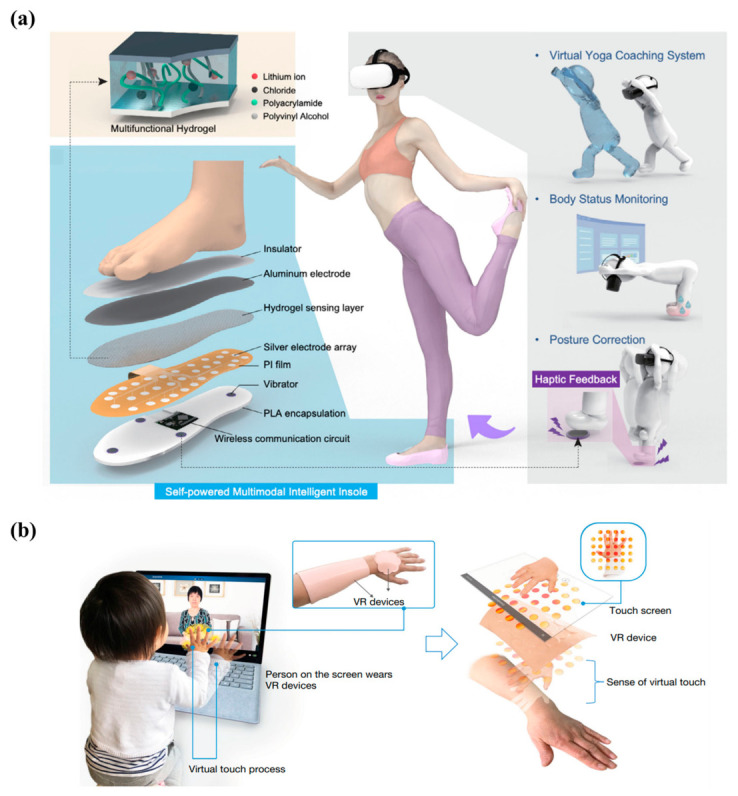
Virtual Reality. (**a**) AI-enhanced multifunctional insole sensing system (Reprinted with permission from [97] © John Wiley & Sons, Inc., 2025); (**b**) Skin-integrated wireless haptic interface system (Reprinted with permission from [98] © Springer Nature, 2019).

With the explosion of Metaverse and Extended Reality (XR) technologies, wearable HMI systems are transitioning from unidirectional command input to bidirectional “sensing-feedback” loops. On the input side (Motion Mapping), to address the “legless avatar” problem and motion distortion in the Metaverse, Wang et al. [97] presented an AI-enhanced multifunctional insole sensing system (AEIS) that leverages ionic hydrogel-based self-powered sensors to create an immersive, metaverse-enabled yoga coaching platform (Figure 14a). By integrating a 32-channel hydrogel sensing array capable of simultaneously monitoring plantar pressure, temperature, and sweat with a customized wireless circuit and embedded haptic vibration units, the system forms a closed-loop, user-centered virtual training framework. The AI engine, employing a ResNet-18 model for yoga posture recognition (98.33% accuracy) and a hybrid model selector for imbalance detection (90.06% accuracy), works in tandem with a random forest regression model trained on expert coach data to predict personalized stability parameters. These real-time assessments are visually conveyed through a metaverse-based yoga coach avatar and a digital twin of the user, while directional vibro-haptic feedback is triggered to physically alert users to improper plantar pressure distributions. This closed-loop paradigm, which merges intelligent multifunctional sensing, AI-driven performance analysis, immersive virtual coaching avatars, and real-time haptic intervention, addresses some limitations of conventional VR training systems, establishing an interactive hardware foundation for deeply engaging, face-to-face-like digital twin experiences and next-generation metaverse healthcare. On the output side (Haptic Feedback), Yu et al. [98] developed a skin-integrated, wireless haptic interface system designed for virtual and augmented reality (VR/AR) applications (Figure 14b). This platform utilizes a thin, soft, and flexible architecture comprising a collection of electronic components and mechanical actuators that conform to the skin’s topography. A defining technical innovation of this work is its battery-free operation, enabled by near-field communication (NFC) protocols for simultaneous wireless power delivery and signal control. The system translates digital information into localized mechanical vibrations, providing users with a tactile dimension in scenarios such as virtual social interactions, prosthetic control, and sensory substitution for rehabilitation. By eliminating the need for bulky batteries and restrictive wiring, this skin-compatible haptic technology provides a robust hardware foundation for bidirectional HAR systems, enabling a fully immersive and energy-autonomous interaction paradigm within the Metaverse.

### 4.4. Behavior Monitoring

The ultimate objective of HAR is to comprehend complex ADL within unconstrained environments. By incorporating environmental context and physiological states, multifunctional sensors reduce the semantic ambiguity of unimodal motion data, achieving an improved understanding of behavioral intent and situational context.

#### 4.4.1. Fall Detection

**Figure 15:**
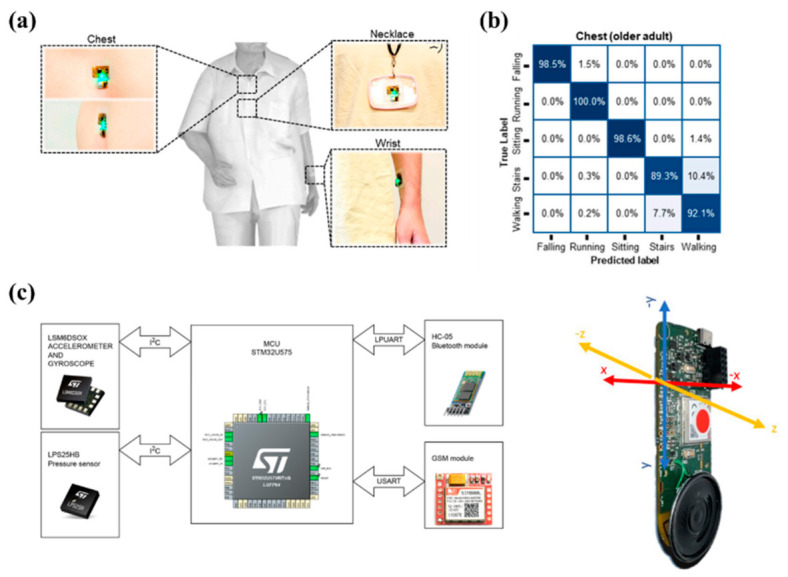
Fall Detection. (**a**) Skin-patch fall monitoring system integrated with IMU; (**b**) Fall detection confusion matrix (Reprinted under a Creative Commons Attribution 4.0 International License from [99] © MDPI, 2023); (**c**) Embedded edge terminal for fall detection (Reprinted with permission from [100] © Institute of Electrical and Electronics Engineers, 2024).

Addressing the critical demand for fall detection in aging societies, wearable HAR systems are evolving toward greater ergonomic adaptability and architectural robustness. Lee et al. [99] conducted an experimental study involving a skin-wearable electronic patch integrated with a miniature inertial measurement unit for continuous fall monitoring among older adults (Figure 15a). A significant technical advancement of this work is the deployment of a sophisticated deep learning framework, which synergizes Convolutional Neural Networks (CNN) for spatial feature extraction with Gated Recurrent Units (GRU) for temporal sequence modeling. This hybrid architecture captures the transient kinematic signatures of various fall types while distinguishing them from ADL. During extensive validation with elderly participants, the system demonstrated high diagnostic performance, achieving a sensitivity of 99.1% and a specificity of 99.4%. By merging the mechanical advantages of skin-conformable electronics with the robust classification power of recurrent neural networks, this paradigm provides a reliable and comfortable hardware-software solution for real-time elderly safety monitoring and emergency intervention (Figure 15b). Addressing the computational constraints of traditional wearables, Campanella et al. [100] introduced a novel embedded deep learning sensor specifically optimized for edge computing in fall detection. This device achieves sophisticated multifunctional data fusion by integrating a pressure sensor alongside a 3-axis gyroscope and a 3-axis accelerometer (Figure 15c). By processing these multi-source kinematic and barometric signals locally, the embedded model operates in real-time with low computational complexity. Crucially, the system not only accurately discriminates between standard falls and routine ADL but also identifies syncope (fainting) episodes as a critical, often-overlooked precursor to sudden falls. Validated against both proprietary and public (SisFall) datasets, this edge-AI paradigm demonstrates that highly accurate, continuous fall surveillance can be achieved with low-cost embedded hardware, eliminating cloud latency and providing a highly responsive digital safeguard for vulnerable populations.

#### 4.4.2. Emotion Recognition

**Figure 16:**
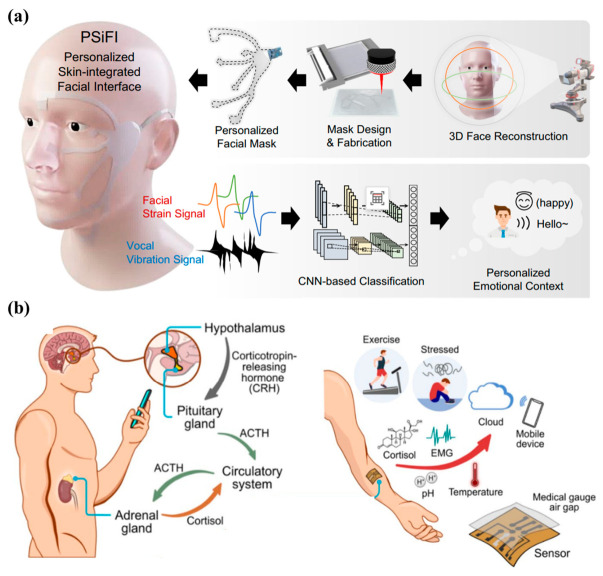
Emotion Recognition. (**a**) Personalized skin-integrated facial interface (PSiFI) (Reprinted under a Creative Commons Attribution 4.0 International License from [102] © Springer Nature, 2024); (**b**) Hybrid wearable physicochemical sensor suite for EMG and sweat cortisol monitoring (Reprinted under a Creative Commons Attribution 4.0 International License from [103] © Springer Nature, 2024).

As HAR advances toward higher-level contextual awareness, precisely quantifying affective states (emotion) and mental stress has become a key challenge for empathic HCI [101]. Targeting the multifunctional expression of explicit emotions during social interaction, Lee et al. [102] developed a personalized skin-integrated facial interface (PSIFI) system for encoding comprehensive multifunctional emotional information. This self-powered, transparent, and highly stretchable platform features an innovative bidirectional triboelectric strain and vibration sensor. Notably, this singular architectural design enables the simultaneous acquisition of both non-verbal expression data (facial muscle strain) and verbal data (vocal cord vibrations) (Figure 16a). Fully integrated with a wireless data processing circuit and empowered by machine learning algorithms, the PSIFI system achieves real-time, high-accuracy emotion recognition. Notably, it effectively decodes complex human affects even when visual cues are completely occluded by facial masks. This paradigm of fusing verbal and non-verbal kinesthetic data via conformal epidermal sensors broadens the capabilities of HAR, laying an integrated hardware foundation for advanced affective computing and empathetic human–machine interactions. Complementing this, to quantify implicit mental stress, Hossain et al. [103] developed a hybrid wearable physicochemical sensor suite, that uniquely fuses EMG, sweat cortisol, and skin physical sensors for non-invasive, real-time human emotion recognition (Figure 16b). This flexible epidermal patch captures muscle tension dynamics via EMG electrodes while simultaneously tracking sweat cortisol—a key neuroendocrine marker of emotional arousal—and corrects cortisol readings through on-board pH and temperature sensors. By cross-validating the immediate neuromuscular responses with the temporally delayed hormonal cascade, the system captures complementary arousal timelines and decouples emotionally induced physiological changes from pure physical exertion. This multifunctional emotion-sensing strategy significantly expands wearable affective computing capabilities, enabling continuous, multi-dimensional assessment of internal emotional states beyond conventional activity or stress monitoring.

## 5. Challenges

Despite breakthroughs in material innovation and structural design, transitioning wearable multifunctional sensors from “laboratory-grade prototypes” to ubiquitous “in-the-wild” deployments confronts severe systemic hurdles spanning fundamental physics to system-level integration.

### 5.1. Signal Decoupling and High-Fidelity Acquisition

A paramount bottleneck for multifunctional HAR sensors lies in achieving signal decoupling and fidelity under unconstrained dynamic environments [104]. Although architecture-level integration mitigates some interference via physical isolation, the vast majority of flexible active materials (e.g., MXene, Laser-induced graphene) exhibit intrinsic cross-sensitivity, where electrical properties are simultaneously modulated by mechanical deformation and thermal fluctuations [105,106]. This physical coupling results in a scenario where high-amplitude motion artifacts spectrally overlap with and mask weak physiological bio-potentials (e.g., ECG, EEG), severely degrading the Signal-to-Noise Ratio (SNR) during vigorous activities [107,108]. Consequently, it is imperative to develop materials with low cross-sensitivity and circuit architectures capable of source-level artifact suppression to extract high-fidelity multidimensional information in complex scenarios.

### 5.2. Long-Term Robustness and Bio-Interfacial Stability

Long-term mechanical robustness and bio-interfacial stability are prerequisites for achieving clinical-grade reliability [109]. While existing micro-crack or nanowire-based structures offer high sensitivity [110,111], they are often plagued by fatigue failure and signal hysteresis after tens of thousands of stretching cycles [112]. Furthermore, the sensing interface is inherently dynamic: sweat accumulation and biofouling can markedly alter the electrode–skin impedance, while any resulting skin irritation may reduce user compliance, collectively inducing measurement drift and progressively degrading accuracy over time [113]. Therefore, future research must focus on advanced encapsulation technologies that balance high moisture permeability (breathability) with robust hydrophobicity and anti-biofouling properties, ensuring stable operation in all-weather environments without sacrificing mechanical compliance.

### 5.3. Individual Variability and Adaptive Calibration

Inter-subject variability is a major challenge for generalized models. This variability spans anatomical features (limb size, muscle density), skin properties (impedance, sweat gland distribution), kinematic patterns (gait, gesture habits), and physiological baselines. As a result, population-based models often fail when applied to new, unseen individuals [114,115]. This “one-size-fits-all” approach often leads to substantial measurement bias and degradation in recognition accuracy. To address this, future systems must integrate personalized calibration protocols and adaptive learning algorithms (e.g., Domain Adaptation, Few-Shot Learning). These capabilities would allow devices to optimize parameters and models online for specific users, thereby overcoming the “domain shift” caused by individual heterogeneity [116].

### 5.4. Energy Autonomy and Efficiency

At the system level, energy autonomy and power budget constraints fundamentally limit the sustainability of multifunctional sensing [117]. As the number of sensing modalities increases, the power consumption associated with continuous data sampling, processing, and transmission escalates exponentially [118]. Although self-powered technologies like TENGs offer promising avenues [119], their intermittent power output under low-frequency human motion often falls short of the budget required for high-frequency wireless telemetry [120]. Consequently, future architectures must evolve toward neuromorphic computing or event-driven sensing paradigms (e.g., Spiking Neural Networks) [121,122]. By processing information at the edge and transmitting only salient “events” rather than raw data streams, these systems can achieve extreme energy efficiency optimization at the architectural level.

### 5.5. Deep Fusion and Explainable AI

Ultimately, the depth of heterogeneous data fusion and model interpretability dictate the translational value of HAR systems. Facing the vast spatiotemporal disparity between physical, physiological, and biochemical data, current “black-box” deep learning paradigms often lack the clinical explainability required for medical decision-making [123,124]. There is an urgent need to develop efficient fusion models capable of handling asynchronous spatiotemporal features (e.g., Cross-modal Transformers) and to integrate biomechanical priors into deep learning networks. Such Physics-Informed Machine Learning (PIML) approaches would ensure that recognition results are not only precise but also mechanistically interpretable, truly empowering the next generation of intelligent medical and HCI systems [125].

## 6. Conclusions and Outlook

This review systematically delineates the evolutionary trajectory of wearable multifunctional sensors for HAR. Sensor technology is evolving from discrete system assembly and monolithic fusion to intrinsically multifunctional materials. This shift moves beyond simple functional stacking toward deep structural and functional integration. The incorporation of multifunctional fusion technologies not only mitigates the perception blind spots of unimodal systems in complex dynamic environments but also facilitates multidimensional information synergy—such as correlating kinematic patterns with physiological biomarkers—thereby providing robust, depth-resolved data support for medical rehabilitation, sports science, and HCI.

Looking forward, the evolution of wearable multifunctional sensors will transcend simple functional addition or performance optimization, advancing toward a novel paradigm characterized by intelligent fusion, autonomous adaptation, and human-centric integration. This field is undergoing changes, merging materials science, electronics, information theory, and biomedicine. These systems are poised to evolve into autonomous, energy-self-sufficient, and reliable intelligent sensing interfaces. Ultimately, they may advance our comprehension of human behavior and health and serve as an enabling technology to support changes in how we interact with and converge into the digital world.

## Figures and Tables

**Table 1:** Comparison of three multifunctional integration strategies.

Feature	Architecture-Level Integration	Device-Level Monolithic Integration	Material-Level Intrinsically Multifunctionality
Core Philosophy	Physical isolation of functional units	Co-located fabrication on single chip/substrate	Single material with orthogonal responses to stimuli
Integration Density	Low-Medium	Medium-High	Extremely High
Signal Decoupling Method	Hardware-level physical isolation	Hardware + Algorithm	Algorithm-based
Key Advantage	High fidelity, independent optimization	Extreme compactness, high spatiotemporal consistency	Ultimate miniaturization, bio-mimetic potential
Main Limitation	Larger footprint, complex assembly	Complex fabrication, risk of residual crosstalk	Requires deep understanding of physics/chemistry
Representative Sensing Modalities	Pressure, temperature, GSR, ECG, EMG	3D force, strain, temperature, proximity	Strain-temperature, pressure-magnetic

**Table 2:** Performance summary of representative wearable multifunctional sensors for HAR.

Integration Strategy	Detection Targets	Sensitive Materials	Sensing Mechanism	Key Performance Parameters	Wireless/Self-Powered	Supported HAR Tasks	Recognition Accuracy	Ref.
Architecture-Level (Lateral)	Sitting pressure (<100 kPa), skin temperature, GSR	Cr/Au (nanocracks), NTC thermistor, Parylene-C	Piezoresistive, thermoelectric, electrochemical impedance	<100 kPa range, low hysteresis, high linearity	NFC, battery-free	Wheelchair pressure monitoring, pressure ulcer prevention	N/A	[16]
Architecture-Level (Vertical)	Facial pressure, sEMG	Porous PVDF, PVA/BMMICl IL, Ag dry electrodes	Capacitive (EDL), bioelectrical acquisition	5 Pa detection limit, 16.8 ms response; sEMG matches commercial electrodes	Bluetooth (external power)	Facial expression recognition, robotic arm control	93.8% (1D-CNN)	[26]
Architecture-Level (System-Level)	Pressure, humidity, temperature, respiration, BCG	rGO-coated PDMS sponge (dual-gradient porous)	Piezoresistive, capacitive, thermoelectric	2.6× higher pressure sensitivity; 5-tier moisture detection	RFID, battery-free	Multi-site physiological monitoring, patient care	N/A	[29]
Device-Level Monolithic	Normal pressure, shear force, tensile strain	Au piezoresistors, graded-modulus polymers	Piezoresistive (3D biomimetic microstructures)	0–80 kPa linear range; ~0.1 mm spatial resolution; 10 k cycles stable	Wired (prototype)	Tactile localization, prosthetic perception	N/A	[21]
Material-Level Intrinsically Multifunctional	Strain, strain rate, temperature	Tilt-grown Te nanowire network	Piezoelectric (AC), thermoelectric (DC)	Single active layer; 225.1 μV·K^−1^ temp sensitivity	Self-powered (piezo/thermo effect)	Joint motion monitoring, HCI	N/A	[50]
Material-Level Intrinsically Multifunctional	Magnetic field, mechanical force, humidity	SA/PVA/glycerol hydrogel, magnetic particles	Capacitive (mag/humidity), piezoresistive (force)	8 k cycles stable; no signal crosstalk	Wired (prototype)	Sign language recognition, multimodal HCI	99.17% (CNN-GRU)	[55]

**Table 3:** Comparison of machine learning classifiers for human activity recognition.

Classifier	Suitable Use	Advantage	Disadvantage	Accuracy	Ref.
SVM	Small-sample datasets, binary classification	Strong generalization, low overfitting, easy mobile deployment	Slow on large datasets, complex multi-class, noise-sensitive	88%	[64]
RF	Multi-class classification, feature evaluation, imbalanced data	Robust to overfitting, handles high-dim data, fast training	Poor on small samples, low interpretability, noise-prone	60.6–94.6%	[65]
HMM	Temporal-dependent activities, sequence modeling, online recognition	Explicit temporal modeling, noise-robust, streaming-friendly	Needs large labeled data, strict Markov assumption, high complexity	89.3%	[66]
CNN	Hierarchical feature extraction, indoor/outdoor HAR	High accuracy, scale-invariant, flexible architecture	Prone to overfitting, struggles with complex dynamic activities	97.44%	[67]
RNN	Time series analysis, temporal encoding, sequence modeling	Captures long-range dependencies, flexible sequence modeling	Vulnerable to vanishing gradients, slow on long sequences	Above 90%	[68]
LSTM	Modeling long-term dependency in time series data	Handle long sequences and flexible data size adaptation	Limited capacity and ignores spatial features	64.96–94.86%	[69]
GRU	Sequential data and long-term dependencies	Minimal parameters, and fast convergence	Limited memory capacity	90%, 91%	[70]

## Data Availability

No new data were created.

## Abbreviations

The following abbreviations are used in this manuscript:

1D-CNN	One-Dimensional Convolutional Neural Network
AC	Alternating Current
ADL	Activities of Daily Living
AI	Artificial Intelligence
AR	Augmented Reality
BAT	Bimodal All-Textile
BPS	Bimodal Piezotronic Sensor
CC@PANI-PB	Polyaniline-Prussian Blue Composite
CCTO	CaCu_3_Ti_4_O_12_
CEDS	Capacitive-Electromyographic Dual-Mode Sensor
CNN	Convolutional Neural Network
CNT	Carbon Nanotube
DC	Direct Current
DNN	Deep Neural Network
DoF	Degree of Freedom
ECG	Electrocardiogram
EDL	Electrical Double-Layer
EEG	Electroencephalogram
EMG	Electromyography
FPCB	Flexible Printed Circuit Board
GF	Gauge Factor
GO	Graphene Oxide
GRU	Gated Recurrent Unit
GSR	Galvanic Skin Response
HAR	Human Activity Recognition
HCI	Human–Computer Interaction
HMI	Human–Machine Interaction
HPOF	Hydrogel-Coated PDMS Optical Fiber
HRS	Heart Rate Strap
IMU	Inertial Measurement Unit
IoT	Internet of Things
LIG	Laser-Induced Graphene
Ln-UCNPs	Lanthanide-Doped Upconversion Nanoparticles
MFCS	Multifunctional Flexible Capacitive Sensor
MG-former	Multi-Task Gait Transformer
MLP	Multilayer Perceptron
MTM	Magnetic Tilted Micropillar
NFC	Near-Field Communication
NIMTE	Ningbo Institute of Materials Technology & Engineering
NTC	Negative Temperature Coefficient
PCA	Principal Component Analysis
PDMS	Polydimethylsiloxane
PEDOT:PSS	Poly(3,4-ethylenedioxythiophene):Polystyrene Sulfonate
PIML	Physics-Informed Machine Learning
PPG	Photoplethysmography
PSIFI	Personalized Skin-Integrated Facial Interface
PU	Polyurethane
PW-TENG	Pulp Wool Triboelectric Nanogenerator
RAP	Rehabilitation Assessment Platform
rGO	Reduced Graphene Oxide
SAP	Superabsorbent Polymer
SEMG	Surface Electromyography
SLR	Sign Language Recognition
SNR	Signal-to-Noise Ratio
SoC	System-on-Chip
TCM	Traditional Chinese Medicine
Te	Tellurium
TENG	Triboelectric Nanogenerator
TFT	Thin-Film Transistor
VR/AR	Virtual Reality/Augmented Reality
XR	Extended Reality
